# Synchronous double primary squamous cell carcinoma and adenocarcinoma of the extrahepatic bile duct: a case report

**DOI:** 10.1186/s13256-015-0600-1

**Published:** 2015-05-19

**Authors:** Youngsun Yoo, Seongpyo Mun

**Affiliations:** Department of Surgery, Chosun University, School of Medicine, 365 Pilmun-daero, Dong-gu Gwangju, 501-717 South Korea

**Keywords:** Adenocarcinoma, Bile duct, Squamous cell carcinoma, Synchronous

## Abstract

**Introduction:**

Synchronous double cancers of the bile duct are exceptionally rare. We here report a case of synchronous squamous cell carcinoma and adenocarcinoma of the extrahepatic bile duct.

**Case presentation:**

A 67-year-old Asian man visited our clinic complaining of jaundice and dark urine. Direct hyperbilirubinemia and an elevated cancer antigen 19–9 level were detected. Preoperative abdominal computed tomography and positron emission tomography showed two masses at the bifurcation of the common hepatic duct and at the distal common bile duct. After biliary drainage, we performed radical pylorus-preserving pancreaticoduodenectomy, without resection margin involvement. Pathological findings revealed that the proximal lesion was a squamous cell carcinoma and that the distal lesion was an adenocarcinoma. Both cholangiocarcinomas were confined to the fibromuscular layer, and there was no communication between the two tumors. Multiple conglomerated metastatic tumors were detected in his liver 3 months after surgery. He died 8 months after diagnosis.

**Conclusions:**

The disease displayed very aggressive behavior and a very poor prognosis. The only chance for long-term survival is treatment with radical resection. Preoperative positron emission tomography-computed tomography is useful in detecting occult cancer.

## Introduction

The most common malignant tumors of the bile duct are adenocarcinomas. Squamous cell carcinomas are very rare with an incidence of less than 1.4% according to Funakawa *et al.* [1] Synchronous double primary cancers have occasionally been reported in the literature: they have usually been located at the gall bladder and the bile duct. To date, only several cases of double primary cancers of the extrahepatic bile duct have been reported. All previous cases were found to be double adenocarcinomas (Table 1). To the best of our knowledge, we here report the first case of synchronous primary squamous cell carcinoma and adenocarcinoma of the extrahepatic bile duct. We also describe previous cases and review the associated literature. In addition, we discuss the clinical characteristics of double primary cancers, including the efficacy of chemotherapy, radiation therapy, optimal management, and prognosis.

**Table 1:** **Synchronous double cancer of extrahepatic bile duct**

**Author**	**Ogawa** ***et al*** **.** [9]	**Bedoui** ***et al*** **.** [10]	**Present study**
**Year**	2001	2011	2013
**Age (years)**	69	67	67
**Sex**	Male	Female	Male
**Site of cancer 1**	Middle CBD	Distal CBD	Common hepatic duct
**Site of cancer 2**	Distal CBD	Distal CBD	Distal CBD
**Operation**	PPPD	PPPD	PPPD
**Pathology of cancer 1**	Adenocarcinoma	Adenocarcinoma	Squamous cell carcinoma
**Pathology of cancer 2**	Adenocarcinoma	Adenocarcinoma	Adenocarcinoma
**Chemotherapy**	NR	NR	None
**Prognosis**	NR	Alive 4 months	Alive 8 months

Abbreviations: CBD, common bile duct; NR, not reported; PPPD, pylorus-preserving pancreaticoduodenectomy.

## Case presentation

**Figure 1:**
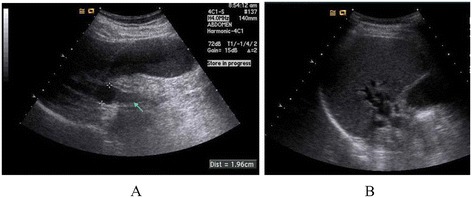
Abdominal ultrasound. **(A)** A tubular mass in distal common bile duct (arrow) and distended gallbladder and common hepatic duct. **(B)** Dilated intrahepatic bile ducts.

**Figure 2:**
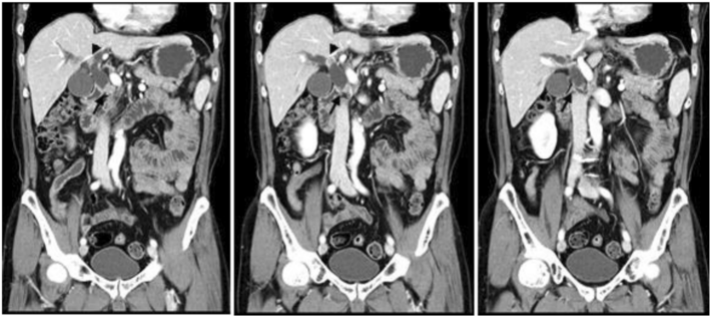
Abdominal computed tomography. Low density mass at common hepatic duct (black arrow head) and distal common bile duct (black arrow) with dilatation of intrahepatic duct and gall bladder.

**Figure 3:**
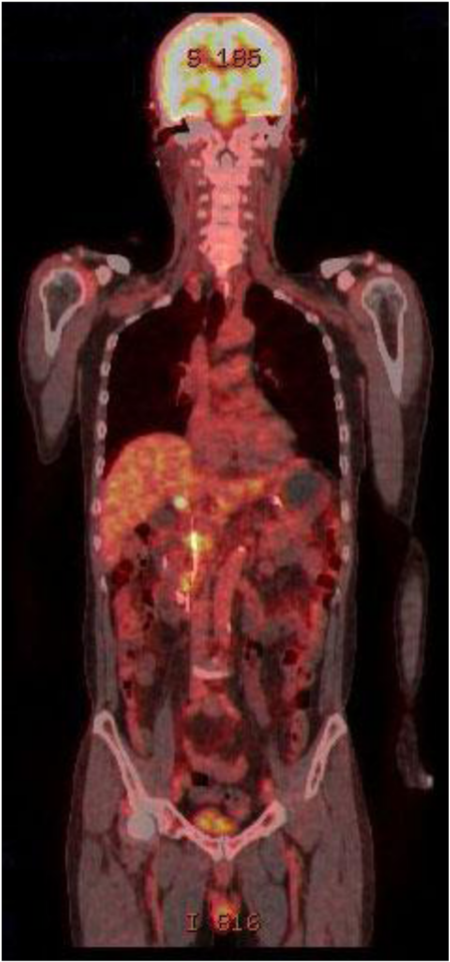
Positron emission tomography. Two hypermetabolic lesions were detected. Maximum standardized uptake value of the lesion of common hepatic duct and distal common bile duct was 8.5 and 7.1 respectively. An endoscopic nasobiliary drainage catheter was placed preoperatively.

**Figure 4:**
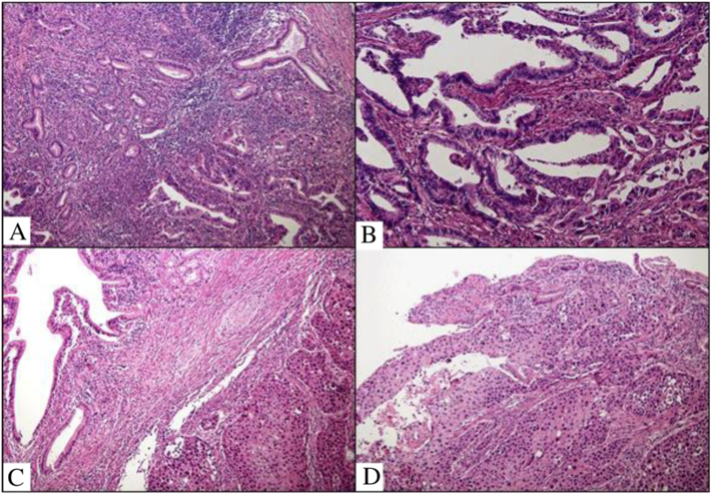
Histopathologic findings. Adenocarcinoma of common bile duct (bottom) adjacent to reactive ductular proliferation (top) **(A)**, which infiltrated into fibromuscular layer with multiple tumor emboli **(B)**. Separate lesion showing well-differentiated squamous cell carcinoma. The tumor was observed adjacent to dilated bile ducts **(C)** and surface epithelium **(D)**.

A 67-year-old Asian man presented to our hospital with yellow skin and dark urine. At first, he found his eyes were yellow 1 month ago. Being yellow extended to his face and trunk which was accompanied by dark urine. He denied any fever, chills or abdominal pain. During the past 6 months, he had lost 7kg of weight. His past medical history was remarkable for total knee replacement 5 years ago. Vital signs were within normal limits. A physical examination revealed a chronically ill appearance and jaundice. There was no evidence of abdominal distension, palpable mass, or organomegaly. His laboratory test results were as follows: aspartate aminotransferase, 260U/L (normal range 5 to 40); serum glutamic-pyruvic transaminase, 567U/L (normal range 5 to 40); gamma-glutamyl transferase, 1504U/L (normal range 16 to 73); total bilirubin, 20.3mg/dL (normal range 0.2 to 1.1); direct bilirubin, 13.7mg/dL (normal range 0 to 0.6); alkaline phosphatase, 924U/L (normal range 42 to 128); and cancer antigen 19–9 (CA19-9), 125.1U/mL (normal range 0 to 33). Abdominal ultrasonography revealed a tubular mass in the distal common bile duct (CBD), dilated intrahepatic bile ducts, and a distended gallbladder (Figure 1). An abdominal computed tomography (CT) scan showed a low-density mass at the common hepatic duct and the distal CBD with dilatation of the intrahepatic duct and his gall bladder (Figure 2). Positron emission tomography (PET) revealed two tumors: one proximal tumor with a maximum standardized uptake value (SUV) of 8.5 and a distal tumor with a maximum SUV of 7.1 (Figure 3). We concluded that the tumors were synchronous double primary cholangiocarcinomas and decided to perform pancreaticoduodenectomy. There was no evidence of anomalous pancreaticobiliary duct union in CT or endoscopic retrograde cholangiopancreatography. In the operative fields, there was no evidence of distant metastasis. Two cholangiocarcinomas were removed with negative surgical margin. The cut surface revealed a gray-white colored, irregularly elevated, firm mass with ulceration in the distal CBD, measuring 17×15mm. On microscopic examination we found a moderately differentiated adenocarcinoma which invaded the fibromuscular layer of the CBD (stage T1b), metastasized to one regional lymph node (N1), and showed multiple lymphovascular tumor emboli. In addition, another mass was present just below the bifurcation of his hepatic duct. It was gray-white in color and measured 15×10mm. Sections from this specimen revealed a well-differentiated squamous cell carcinoma confined to the fibromuscular layer (stage T1b, Figure 4). In addition to the distinct localization of these tumors, there was no transitional area between the two lesions and no intermingled histological features favoring a diagnosis of adenosquamous cell carcinoma. Taking all of these findings into consideration, the diagnosis was synchronous double adenocarcinoma and squamous cell carcinoma of the bile duct. The patient displayed a good clinical course and was discharged 23 days after surgery. However, 3 months later, multiple liver metastases were detected. He refused treatment and died 8 months after the operation.

## Discussion

The annual incidence of perihilar cholangiocarcinoma (PHC) in the USA is about 1 to 2 per 100,000. Patients with PHC typically present with painless jaundice. Imaging such as CT, magnetic resonance imaging (MRI) or PET scan is required for evaluation for distant metastases and resectability. Resectability depends on the biliary extent of the tumor and involvement of the portal vein and hepatic artery. Preoperative biliary drainage is recommended because of the risk of liver failure associated with major hepatectomy in the setting of bile duct obstruction. Biliary drainage of the presumed remnant liver is preferred and can be done percutaneously or endoscopically. A brushing biopsy is typically performed at the time of biliary drainage, but is often indeterminate. Pathologic confirmation of malignancy is not a requirement to proceed with surgical resection. Portal vein embolization is generally recommended in patients with a presumed remnant liver of less than 40%. At the time of presentation, most patients with PHC have metastatic or locally advanced disease and consequently do not benefit from a resection. The median overall survival for un-resected patients is about 12 months [2].

Anomalous pancreaticobiliary ductal union (APBDU) is frequently associated with choledochal cysts and the incidence of malignancy in choledochal cysts ranges from 2.5 to 26%. APBDU is classified into two types: the pancreatico-choledochal (P-C) type, in which the main pancreatic duct enters the CBD, and the choledocho-pancreatic (C-P) type, where the CBD enters the main pancreatic duct. The incidence of P-C type ductal junctions is higher in patients with cancer of the gallbladder, while the incidence of C-P type junctions is higher in patients with carcinoma in congenital cystic dilatation. The incidence of gallbladder cancer associated with APBDU is reported to range from 16.7 to 18.3%. Neoplastic development in the bile duct of patients with APBDU evolves through a multistep process associated with hyperproliferation and genetic alterations. In one study, the *K-ras* mutation was not observed at any site of cancer. However, epithelial cells in both cancerous areas showed positive Ki-67 and it might have caused another signal regulation apart from the *K-ras* gene [3]. The genetic difference between cholangiocarcinoma with APBDU and without APBDU would give us information about the carcinogenesis of the two different types.

Warren and Gates defined multiple primary cancers as follows: each tumor must present a definite picture of malignancy, each tumor must be distinct, and the probability that one is a metastasis of the other must be excluded [4]. The case described here meets all of these requirements. The two tumors were definitively determined to be adenocarcinoma and squamous cell carcinoma. There was no communication between the tumors. In addition, there was no evidence of metastatic metaplasia. To the best of our knowledge, this report is the first to describe a case of synchronous double primary adenocarcinoma and squamous cell carcinoma of the extrahepatic bile duct.

The potential origins of squamous cell carcinoma of the biliary tree include transformation of the adenocarcinoma to squamous cell carcinoma, derivation from the metaplastic squamous epithelium of the glandular tissue, derivation from the ectopic squamous epithelium, and derivation from undifferentiated basal cells. In a study focusing on adenosquamous carcinomas of the gallbladder, the growth rate of squamous cell carcinomas exceeded that of adenocarcinomas, suggesting that adenocarcinomas can transform into squamous cell carcinomas [5]. However, this theory is not supported by reports of cases of pure squamous cell carcinoma without adenocarcinoma components. Thus, squamous metaplasia of the biliary mucosa caused by inflammatory irritation or derivation from metaplastic squamous epithelium of glandular tissue has been considered to be the primary mechanism. Ascariasis, clonorchiasis, choledochal cysts, Caroli’s disease, and primary sclerosing cholangitis can be causative diseases. In animal studies, ectopic squamous epithelium was found in the jejunum. In some cases of cholecystitis complicated by gallstones, undifferentiated basal cell layers were detected in the gall bladder. There was also a case report of an undifferentiated basal cell carcinoma of the pancreas or uterus. Based on these reports, squamous differentiation from undifferentiated basal cells resulting in squamous cell carcinoma is a possibility at various sites. In the present case, however, there was no history of recurrent inflammation of the biliary tract. Pathological findings did not reveal squamous metaplasia or glandular components containing squamous epithelium. Therefore, we hypothesize that the mechanism of pathogenesis was derivation from the ectopic squamous epithelium or from undifferentiated basal cells. Unfortunately, there is no method to test this hypothesis.

The clinical features of squamous cell carcinoma of the bile duct are similar to those of adenocarcinoma with possible enhanced aggressiveness. The patient displays symptoms of biliary obstruction such as jaundice, dark urine, itch, and weight loss. Laboratory tests reveal direct hyperbilirubinemia and abnormal liver function. Levels of tumor markers such CA 19–9 and carcinoembryonic antigen can be elevated. The lesion is usually detected preoperatively using ultrasonography, CT, MRI, or endoscopic retrograde cholangiopancreatography. Radical resection is the only option for long-term survival [6]. Preoperative detection of synchronous tumors of the bile duct is not always possible. In addition, benign stricture, parasites, or gallstones can mimic cholangiocarcinoma. If the occult cancer is present in the proximal bile duct, pancreaticoduodenectomy is associated with a high likelihood of tumor cell involvement of the resection margin. In such cases, PET-CT appears to be very useful. PET-CT provides information not only about the malignant potential of the lesion but also about regional and distant metastasis. In the present case, the proximal tumor was not detected by preoperative ultrasonography. However, both CT and PET-CT clearly showed both tumors. A high maximum SUV helped estimate the malignant potential of the proximal lesion [7]. We were able to remove the tumor via en bloc resection, which includes resection of the common hepatic duct at the bifurcation.

Unlike lesions at other sites, squamous cell carcinoma in the bile duct is resistant to radiation therapy. The mean survival time is 3 to 8 months. One of the main concerns is the benefit of preoperative histological diagnosis of squamous cell carcinoma. Gatof *et al*. reported successful histological diagnosis of squamous cell carcinoma of the bile duct using transpapillary cholangioscopic biopsy [8]. Currently, various thin cholangioscopes with diameters of less than 5mm are available, which enables us to attempt cholangioscopic biopsy of the bile duct tumor. However, the benefit of a histological diagnosis of squamous cell carcinoma seems to be small because it does not affect the management plan. There is no report regarding neoadjuvant or adjuvant combined chemoradiation therapy.

Multiple carcinomas in a single organ are known to occur in different parts of the gastrointestinal tract. Multicentric tumors were reported to occur in up to 10.7% of colon cancer patients, 31% of pancreatic cancers patients, and 80% of esophageal cancer patients in one series. However, multiple tumors in the biliary tract are not reported frequently and cases with double cholangiocarcinomas are exceptionally rare. The first case was reported by Ogawa *et al*.; because the two cancers were adenocarcinomas, they were analyzed for loss of heterozygosity (LOH) to clarify whether indeed double primary cancers were present. Both cancers showed LOH regions at 5q, 6q, 9p, 17p, and 18q. The upper cancer showed one additional LOH region at 8p. The presence of one additional LOH region in the upper cancer suggests that it was a metastasis of the lower one. Strictly speaking, the case reported by Ogawa *et al*. does not meet the criteria of double primary cancers [9]. Bedoui *et al*. reported another case of double adenocarcinomas of the CBD. The two cancers were located distal to the CBD. Both were found to be adenocarcinoma. There was no communication between the cancers and no periductal lymphatic spread. The authors therefore concluded that the adenocarcinomas were double primary cancers [10]. Double primary cancers can be defined only when the possibility of metastasis is excluded. Although there was no connection between the cancers, this does not exclude the possibility of metastasis. Genetic analysis should have been performed, as in the case reported by Ogawa *et al*. In the present case, LOH analysis was not indicated because the two cancers consisted of different cell types: squamous cell carcinoma and adenocarcinoma. However, genetic analysis would provide more information regarding the carcinogenesis of squamous cell carcinoma of the bile duct.

In one study, intraductal neoplastic components showed diffuse staining for c-Met localized in the cell membrane, whereas 10 of the 14 cases lacked expression of c-Met in the corresponding invasive components. In four cases, invasive components composed of undifferentiated carcinoma (n=2), signet-ring cell carcinoma (n=1), and squamous cell carcinoma (n=1) showed nuclear or perinuclear staining for c-Met. Conversely, 11 of the 13 cases did not express Ki-67 in intraductal components, whereas corresponding invasive components showed diffuse nuclear staining in all 14 cases. A comparison of the patterns of expression between c-Met and Ki-67 at the boundary between intraductal and invasive components clearly demonstrated the complementary expression of c-Met and Ki-67. The results suggested that c-Met is involved in early events of carcinogenesis and Ki-67 is involved in the formation of invasive carcinoma [11].

## Conclusions

To the best of our knowledge, we report the first case of synchronous double squamous cell carcinoma and adenocarcinoma of the extrahepatic bile duct. The disease displayed very aggressive behavior and a very poor prognosis. The only chance for long-term survival is treatment with radical resection. Preoperative PET-CT is useful in detecting occult cancer.

## Consent

Written informed consent was obtained from the patient’s next-of-kin for publication of this case report and any accompanying images. A copy of the written consent is available for review by the Editor-in-Chief of this journal.

## Abbreviations

APBDU: Anomalous pancreaticobiliary ductal union
CA 19–9: Cancer antigen 19–9
CBD: Common bile duct
CT: Computed tomography
LOH: Loss of heterozygosity
MRI: Magnetic resonance imaging
P-C: Pancreatico-choledochal
PET: Positron emission tomography
PHC: Perihilar cholangiocarcinoma
SUV: Standardized uptake value
